# Application of Chemical Ionization in the Analysis of Ethylphosphonic Acid Derivatives

**DOI:** 10.1021/jasms.3c00392

**Published:** 2024-03-04

**Authors:** Maciej Boczkowski, Stanisław Popiel, Jakub Nawała

**Affiliations:** Institute of Chemistry, Military University of Technology, Kaliskiego 2, Warsaw 00-908, Poland

**Keywords:** organophosphorus compounds, ethylphosphonic acid derivatives, gas chromatography, chemical ionization, mass spectrometry

## Abstract

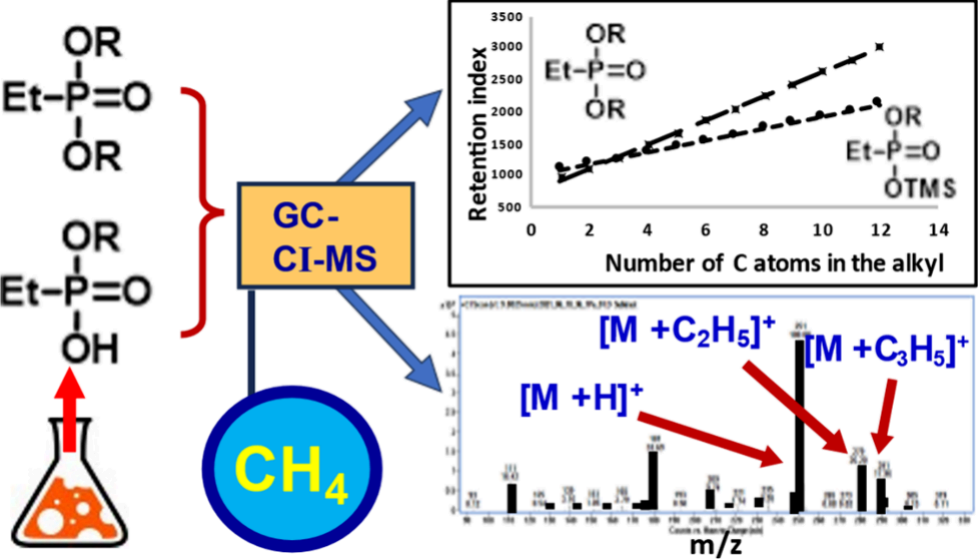

The microsynthesis of 32 dialkyl derivatives of ethylphosphonic acid and the same number of monoalkyl derivatives was carried out to perform comparative studies using gas chromatography combined with mass spectrometry in chemical ionization mode which is one of the analytical techniques recommended by the Organisation for the Prohibition of Chemical Weapons (OPCW). The huge number of possible representatives makes it difficult to have complete spectral libraries of all substances in this class. Therefore, we decided to synthesize and instrumentally analyze only representatives of the selected series of homologues in this work. The analysis of the obtained results allowed us to find the rules for predicting mass spectra and the factors determining the retention parameters. Symmetrical diesters and monoesters of ethylphosphonic acid were selected for this study. During the conducted experiments using chemical ionization with methane as the reaction gas, protonated analyte molecules with high relative intensities were obtained; in many cases, these are base peaks in the spectrum. The obtained results allow grouping of the synthesized compounds depending on the introduced alkyl substituent. Retention data of the tested analytes were collected during the research by using electron ionization. The retention parameters of the tested compounds from each homologous series were also summarized and compared. Chemical Warfare Agents (CWA) analysis continues to be an important issue, especially in the context of the regular Proficiency Tests organized by the OPCW for identifying chemical compounds that are of interest to the Chemical Weapons Convention. Five compounds were synthesized whose spectra were not available in EI mass spectral libraries, and their retention indices were unknown. The identification of these substances was supported by the CI mass spectra and retention data, using previously developed relationships. Therefore, it is reasonable to conclude that the research method used is useful and effective.

## 1 Introduction

Adopted in 1992 by resolution (A/RES/47/39),^1^ signed in 1993 and implemented in 1997, “Convention on the Prohibition of the Development, Production, Stockpiling, and Use of Chemical Weapons and on their Destruction” was and still is a big step toward building a world free from the threats associated with the massive use of highly toxic substances.^2^

One of the outcomes of signing the Convention was the establishment of the Organisation for the Prohibition of Chemical Weapons (OPCW), the implementing body for the Chemical Weapons Convention (CWC). One of its missions was the launch of the Proficiency Tests for a global network of designated laboratories. Initiating this process required the definition of many technical issues. During the 54 editions of the tests, both the requirements for the recommended analytical techniques and reporting of the results have evolved. However, “spectrally rich” methods are still preferred. One of the class of analytical techniques that the OPCW Technical Secretariat (TS) recommends for use in testing compounds related to the Convention is gas chromatography combined with chemical ionization (CI) mass spectrometry.^3^

The first published mass spectra of Chemical Warfare Agents (CWA) obtained by chemical ionization, developed in a tabular version using methane, ethylene, and isobutane as reaction gases, were published in 1979.^4^ A team of Canadian scientists from the Defense Research Establishment Suffield published a series of papers on CWA organophosphorus mass spectrometry;^5,6^ the main focus was on tabun and its impurities.^7−9^ Research on the chemical ionization of alkylphosphonic acid derivatives was also conducted in other countries.^10,11^ This technique is now often used as a supplement to classical electron beam ionization (EI) in research on “conventional compounds”.^12−14^

An essential part of the Convention is the 3 Schedule of chemical compounds, including banned and controlled substances. The schedules of the organophosphorus compounds are particularly extensive. One of them, 2.B.4, is a formally open set of compounds. It is defined as “Chemicals, except for those listed in Schedule 1, containing a phosphorus atom to which is bonded one methyl, ethyl or propyl (normal or iso) group but not further carbon atoms”.^15^ This schedule includes, among others: alkylphosphonic dichlorides, i.e., direct precursors for the production of, e.g., CWA series G, which are listed in the Convention as Schedule 1.A.1. Schedule 2.B.4 also includes products of direct environmental decomposition of these substances, monoesters of alkylphosphonic acids, which in the next stage degrade to alkylphosphonic acids, which are stable and persistent compounds in the environment. They can serve as markers of CWA usage, even after a long time. It should be noted that these substances cannot be analyzed directly by GC-MS due to their high boiling points and the presence of polar hydroxyl groups. The solution is their conversion to volatile derivatives by derivatization. In the case of environmental samples, conversion to silyl esters is recommended.^16^ In the case of biomedical samples, alkylation with perfluorinated reagents is used.^17^ Recommended procedures for conducting the derivatization reaction have been developed.^18^ These compounds in unchanged form (without derivatization) can be analyzed by liquid chromatography with selected detectors^19−21^ or gas chromatography using a CP-FFAP column.^22^ This column was deployed by the producer to analyze the polar compounds, such as free fatty acids, alcohols, or organic acids. The FFAP stationary phase is polyethylene glycol modified with acid to provide an inert column that can ensure the demanding analysis of polar compounds. Therefore, it allows for the analysis of alkylphosphonic acids without derivatization.

The number of possible derivatives of Schedule 1.A was initially estimated at about 500,000.^23^ Later reports gave values of approximately 1,325,000.^18^ Recently, it has been estimated that the number of representatives of Schedule 1.A.1 alone is 2,347,712,^24^ and the number of their derivatives assigned to Schedule 2.B.4 will be the same. The most toxic compounds from Schedule 1.A.1 contain branched alkyls with 4–6 carbon atoms in the ester groups.^25^ Derivatives containing long carbon chains in ester groups are indeed unlikely to be considered mass-produced CWA due to their price, availability, and relatively low toxicity.^18^ However, their use for criminal purposes cannot be ruled out.

The huge number of possible compounds makes it difficult to have complete libraries of spectra of all compounds in these classes. Therefore, it seems reasonable to synthesize and instrumentally analyze selected series of homologues.^26,27^ Elaboration of the obtained results will make it possible to find the rules for predicting the mass spectra of this class of compounds and the factors determining the retention parameters. The acquired knowledge will allow us to predict the properties of their other not yet described in the literature or still unexplored representatives, which will be the aim of this work. Symmetrical diesters and monoesters of ethylphosphonic acid were selected for the study. Safety considerations dictated the choice of these substances, because substances from Schedule 1 are highly toxic.

During the research, undecyl and dodecyl derivatives were also obtained, which, although included in Schedule 2.B.4, cannot be considered as degradation products of CWA from Schedule 1 because compounds with chains of up to 10 carbon atoms are considered such. In this work, they were used to confirm the behavior trend of the *n*-alkyl derivatives. Their spectra and the spectrum of the O-cyclobutyl O-TMS ethylphosphonate are not included in the available libraries (NIST17, OCAD_v24, VGWG_2022, nor WILEY).

To the best of the authors’ knowledge, comprehensive information on the studies of ethylphosphonic acid derivatives using GC-CI-MS has not been published so far.

## 2 Experimental Section

### 2.1 Reagents and Chemicals

The following chemicals compounds all with purity of 96% or more were substrates used for microsynthesis and preparing samples for analysis: methanol, acetonitrile (Chem-Solve, Łódź, Poland); ethanol, dichloromethane (POCH, Gliwice, Poland); 1-propanol, 1-butanol, 1-pentanol, 1-hexanol, 1-heptanol, 1-octanol, 1-nonanol, 1-decanol, 1-undecanol, 1-dodecanol, cyclobutanol, cyclopentanol, cyclohexsanol, cycloheptanol, 2-propanol, 2-butanol, 2-methyl-1-propanol, 2-pentanol, 3-pentanol, 2-methyl-1-butanol, 3-methyl-1-butanol, 3-methyl-2-butanol, 2-hexanol, 3-hexanol, 2-methyl-1-pentanol, 2-ethyl-1-butanol, 4-methyl-2-pentanol, N,O-Bis(trimethylsilyl)trifluoroacetamide (BSTFA), alkane solution (C_7_–C_30_) (Sigma-Aldrich, St. Louis, MO, USA); 2,2-dimethyl-1-propanol, ethylphosphonic dichloride: (Acros Organics, USA); pinacolyl alcohol: (Alfa Aesar, Ward Hill, MA, USA), cyclooctanol (Merck, Darmstadt, Germany); sodium sulfate: (CHEMPUR, Piekary Śląskie, Poland); deionized water with a conductivity of 0,05 μS obtained from a Polwater DL-2-50 deionizer.

### 2.2 Instrumental

Spectral and retention data were obtained using Agilent 7890B gas chromatograph coupled with Agilent 7000C triple-quadrupole mass spectrometer both in EI and CI modes with HP-5MS UI (J & W) capillary column (30 m, 0.25 mm i.d., and 0.25 μm film thickness). The carrier gas was helium 6.0 purity with 1 mL/min flow. For CI experiments in positive mode methane, 5.5 purity was used, with a flow of 20% controlled by the device.

### 2.3 Analytical Method

The analysis was carried out using a temperature program: the chromatographic column was held for 1 min at 40 °C and then heated to 320 °C at a rate of 20 °C/min, sustaining the final temperature of 320 °C for 3 min. The injection temperature was 250 °C in splitless mode with purge flow to split vent 55 mL/min at 0.2 min, transfer line temperature 320 °C, ion source temperature 230 °C for EI and 250 °C for CI, both quadrupole 150 °C, electron energy 70 eV for EI and 150 eV for CI, emission current 35 eV for EI, 145 eV for CI, scan range 40–500 u for EI and 80–600 u for CI. One μL portion of the sample was introduced. For dimer identification, the mass range was 100–900 for CI analysis.

### 2.4 Procedure for Microsynthesis of Ethylphosphonic Acid Derivatives

Due to the fact that most of the analytes selected for research are not commercially available, microsynthesis of dialkyl derivatives and monoalkyl derivatives of ethylphosphonic acid were performed. The general reaction equation for the conducted microsynthesis of symmetrical diesters is as follows:
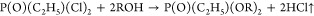
The overall reaction equation for the conducted microsynthesis of monoesters is as follows:
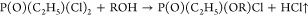

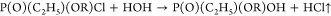
The resulting monoesters were then derivatized with BSTFA:
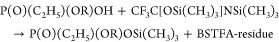


Every derivative was obtained on a microscale in “one pot” synthesis and was not isolated from the postreaction mixture. Into the 5 mL vial was added 0.1 mL of appropriate alcohol mixed with 50 μL of pyridine and 10% solution of ethylphosphonic dichloride in *n*-hexane. The mixture was vortexed and heated at 60 °C for 60 min. After this time, 1 mL of deionized water was added and shaken for 1 min, and then 1 mL of *n*-hexane was added and shaken again for 1 min. After phase separation, the upper phase was collected and dried over anhydrous sodium sulfate. The standard solutions obtained in this way were analyzed by using GC-EI-MS and GC–CI-MS. O-alkyl-O-trimethylsilyl ethylphosphonates were obtained by evaporating 100 μL of postreaction water to dryness in a stream of nitrogen and derivatizing the residue with BSTFA in acetonitrile solution for 30 min and at 60 °C. After derivatization, the reaction mixture was cooled, diluted with dichloromethane, and analyzed by GC-EI-MS and GC-CI-MS.

**Figure 1:**
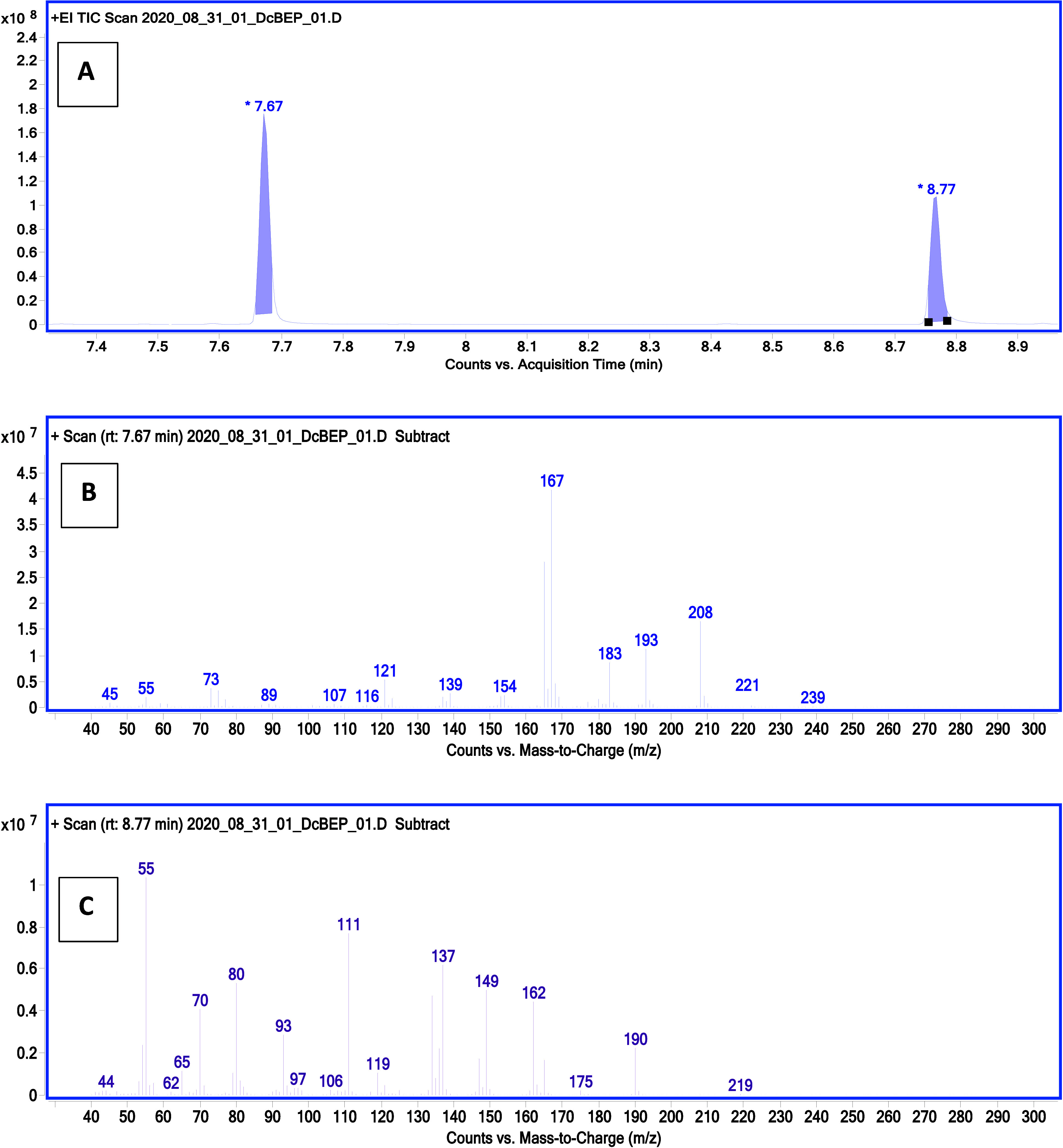
Chromatogram (A) and mass spectra (B, C) showing the analysis of the postreaction mixture after microsynthesis of the dicyclobutyl derivative of ethylphosphonic acid. Compound with a retention time of 7.67 min is *O*-cyclobutyl *O*-trimethylsilyl ethylphosphonate and the compound with a retention time of 8.77 min is *O*,*O*-dicyclobutyl ethylphosphonate.

**Figure 2:**
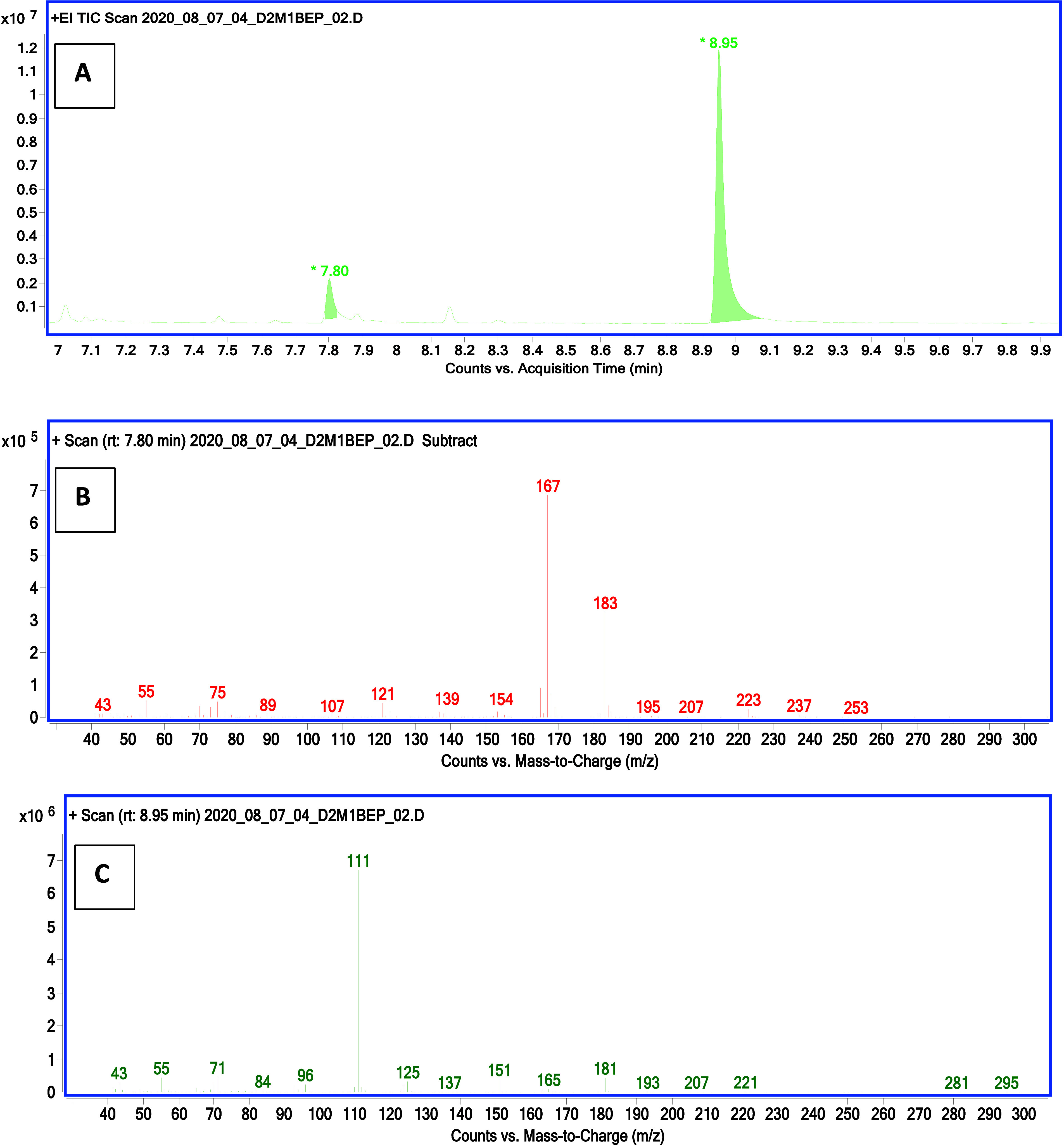
Chromatogram (A) and mass spectra (B, C) showing the analysis of the postreaction mixture after microsynthesis of the di2methyl-1-butyl derivative of ethylphosphonic acid. Compound with a retention time of 7.80 min is O-2methyl-1-butyl O-trimethylsilyl ethylphosphonate, and the compound with a retention time of 8.95 min is O,O-di2methyl-1-butyl ethylphosphonate.

The resulting postreaction mixtures were not purified and analyzed as a mixture. The correctness of the microsyntheses was confirmed using GC-EI-MS. Example chromatograms and mass spectra are shown in Figures 1 and 2.

### 2.5 Health and Safety Section

Safety precautions were taken during synthesis and when handling samples and solutions containing the compounds. When setting up reactions, sampling and preparing analytical samples, and cleanup or decontamination activities, the following personal protective equipment were worn: safety glasses, nitrile gloves, and lab coat. All activities involving synthesis and sample preparation were performed in the laboratory room in a ventilation hood.

## 3 Results and Discussion

### 3.1 Properties of Positive Chemical Ionization (PCI) Spectra

In the EI spectra of synthesized 32 *O*,*O*-dialkyl ethylphosphonates and the same number of *O*-alkyl *O*-trimethylsilyl ethylphosphonates, no molecular ion with significant abundance is observed, which is consistent with the available literature data.^28^ Of course, the exceptions are the dimethyl and diethyl derivatives, whose spectra have a molecular ion (Tables 1 and 2). Therefore, obtaining mass spectra with a molecular ion or a protonated analyte molecule will be of great importance for identification, because the molecular weight of the compound is an additional and always important parameter characterizing a chemical compound. It is worth noting that the OPCW TS recommends in its guidelines providing this parameter when reporting identified analytes in Proficiency Tests.^3^

**Table 1:** Spectral and Retention Data Experimentally Determined for *O*,*O*-Dialkyl Ethylphosphonates

Lp.	R	molecular weight^b^	M^+•^ in EI %	base peak in EI *m*/*z*	[M + H]^+^ in CI *m*/*z*	[M + H]^+^ in CI %	[M + 29]^+^ in CI %	[M + 41]^+^ in CI %	[2M + H]^+^ in CI *m*/*z*	[2M + H]^+^ in CI %	base peak in CI *m*/*z*	characteristic peak in CI (*m*/*z* 111) %	LTPRI
1	methyl	138	7	110	139	100	11	11	277	16	139	0	971
2	ethyl	166	7	111	167	100	22	14	333	17	167	8	1091
3	1-propyl	194		111	195	100	19	18	389	22	195	18	1274
4	1-butyl	222		111	223	100	19	16	445	1	223	25	1459
5	1-pentyl	250		111	251	100	25	17	501	6	251	16	1649
6	1-hexyl	278		111	279	100	29	16	557	5	279	15	1842
7	1-heptyl	306		111	307	100	31	16	613	4	307	12	2036
8	1-octyl	334		111	335	100	31	15	669	1	335	11	2231
9	1-nonyl	362		111	363	100	25	11	725	1	363	9	2427
10	1-decyl	390		111	391	100	26	11	781	1	391	7	2648
11	1-undecyl	418		111	419	100	25	11	837	1	419	8	2821
12	1-dodecyl	446		111	447	100	24	10	893	1	447	5	3010
13	2-propyl	194		111	195	100	3	0	389	20	195	90	1146
14	2-butyl^a^	222		111	223	76	0	0	445	1	111	100	1339
15	2-pentyl^a^	250 T		111	251	51	0	0	501	1	111	100	1495
16	2-hexyl^a^	278 T		111	279	31	0	0	557	1	111	100	1675
17	cyclobutyl	218		55	219	100	6	8	437	7	219	20	1539
18	cyclopentyl	246		111	247	57	0	0	493	5	111	100	1745
19	cyclohexyl	274		111	275	61	0	0	549	3	111	100	1962
20	cycloheptyl	302		111	303	4	0	0	607	1	95	80	2239
21	cyclooctyl	330		111	331	0	0	0	661	1	109	53	2499
22	3-pentyl	250		111	251	26	0	0	501	1	111	100	1514
23	3-hexyl^a^	278		111	279	5	0	0	557	1	83	68	1675
24	2-methyl-1-propyl	222		111	223	100	3	12	445	3	223	73	1373
25	2,2-dimethyl-1-propyl	250		124	251	100	1	8	501	1	251	66	1442
26	2-methyl-1-butyl	250		111	251	100	4	11	501	1	251	71	1568
27	3-methyl-1-butyl^a^	250		111	251	100	24	18	501	1	251	16	1563
28	3-methyl-2-butyl^a^	250 T		137	251	6	0	0	501	1	111	100	1478
29	2-methyl-1-pentyl^a^	278		111	279	100	6	11	557	2	279	75	1736
30	2-ethyl-1-butyl	278		111	279	100	3	9	557	2	279	98	1735
31	4-methyl-2-pentyl^a^	278 T		111	279	11	0	0	557	1	111	100	1498
32	pinacolyl^a^	278 D		137	279	68	0	0	557	1	111	100	1587

a Alcohol with a chiral center.

b D – double peaks in the chromatogram; T – triple peaks in the chromatogram; LTPRI – linear temperature-programmed retention index.

**Table 2:** Spectral and Retention Data Experimentally Determined for *O*-Alkyl *O*-Trimethylsilyl Ethylphosphonates

Lp.	R	molecular weight^b^	M^+•^ in EI %	The base peak in EI *m*/*z*	[M + H]^+^ in CI *m*/*z*	[M + H]^+^ in CI %	[M + 29]^+^ in CI %	[M + 41]^+^ in CI %	*m*/*z* 167 in CI %	*m*/*z* 183 in CI %	[M + 73]^+^ in CI *m*/*z*	[M + 73]^+^ in CI %	base peak in CI *m*/*z*	LTPRI
1	methyl	196	7	181	197	100	23	10	2	2	269	1	197	1105
2	ethyl	210	7	167	211	100	27	13	5	6	283	18	211	1158
3	1-propyl	224		167	225	100	26	15	12	21	297	24	225	1243
4	1-butyl	238		167	239	100	23	14	13	36	311	6	239	1334
5	1-pentyl	252		167	253	100	26	14	12	37	325	28	253	1425
6	1-hexyl	266		167	267	100	27	14	11	36	339	31	267	1522
7	1-heptyl	280		167	281	100	27	14	9	34	353	30	281	1617
8	1-octyl	294		167	295	100	27	13	8	32	367	24	295	1716
9	1-nonyl	308		167	309	100	26	12	7	30	381	12	309	1812
10	1-decyl	322		183	323	100	25	11	5	25	395	6	323	1914
11	1-undecyl	336		183	337	100	25	11	5	24	409	13	337	2010
12	1-dodecyl	350		183	351	100	24	10	5	23	423	7	351	2111
13	2-propyl	224	1	167	225	72	0	6	36	100	297	28	183	1180
14	2-butyl^a^	238		167	239	74	0	0	33	100	311	44	183	1272
15	2-pentyl^a^	252 T		167	253	60	6	3	31	100	325	30	183	1359
16	2-hexyl^a^	266 T		167	267	48	2	1	28	100	339	18	183	1430
17	cyclobutyl	236		167	237	100	14	11	12	40	309	13	237	1368
18	cyclopentyl	250		167	251	54	0	0	30	100	323	31	183	1465
19	cyclohexyl	264		167	265	60	0	0	30	100	337	27	183	1564
20	cycloheptyl	278		167	279	20	0	0	26	100	351	6	183	1690
21	cyclooctyl	292		167	293	6	0	0	25	100	365	1	183	1807
22	3-pentyl	252		167	253	57	0	0	30	100	325	34	183	1364
23	3-hexyl^a^	266		167	267	21	2	0	27	100	339	4	183	1430
24	2-methyl-1-propyl	238		167	239	97	12	15	36	100	311	8	183	1293
25	2,2-dimethyl-1-propyl	252		167	253	100	8	11	31	92	325	76	253	1328
26	2-methyl-1-butyl	252		167	253	93	10	13	35	100	325	24	183	1386
27	3-methyl-1-butyl^a^	252		167	253	100	25	15	14	43	323	27	253	1387
28	3-methyl-2-butyl^a^	252 T		167	253	12	0	0	27	100	325	1	183	1340
29	2-methyl-1-pentyl^a^	266		167	267	93	11	13	33	100	339	26	183	1469
30	2-ethyl-1-butyl	266		167	267	79	9	10	32	100	339	25	183	1471
31	4-methyl-2-pentyl^a^	266 T		167	267	18	1	1	27	100	339	2	183	1389
32	pinacolyl^a^	266		167	267	62	3	0	20	81	339	100	339	1387

a Alcohol with a chiral center.

b T – triple peaks in the chromatogram; LTPRI – linear temperature programmed retention index.

The mass spectrum obtained in the CI mode gives another piece of information that can be used to identify the compound, especially in the case of such classes of substances whose mass spectra in the EI mode are poor, slightly different within homologues, and do not form a molecular peak in this mode (Tables 1 and 2). The use of CI results in the formation of a protonated molecule with much greater relative intensity than the molecular ion formed during EI ionization. This is due to less energy deposited on the ionized molecule, resulting in less fragmentation.^29^ The ion of the protonated molecule is often the base peak in the mass spectrum obtained in chemical ionization mode.

The most common phenomenon during the formation of positive ions in the gas phase is the proton transfer process:^29,30^
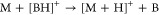


This is also the case when methane is used as the reaction gas and the main reaction ion is [CH_5_]^+^. The molecular weight is confirmed by characteristic adducts. In the case of methane, two additional ions are often observed in the spectrum: [M + C_2_H_5_]^+^ and [M + C_3_H_5_]^+^. They are formed in the process of electrophilic addition.^30^ Methane is one of the most commonly used substances for this purpose.

Admittedly, this reagent is considered a hard reaction gas, i.e., causing relatively high fragmentation. The use of ammonia may in some analytical solutions be more efficient in obtaining protonated molecules.^10^

### 3.2 Methane PCI Spectra of the Tested Compounds

**Figure 3:**
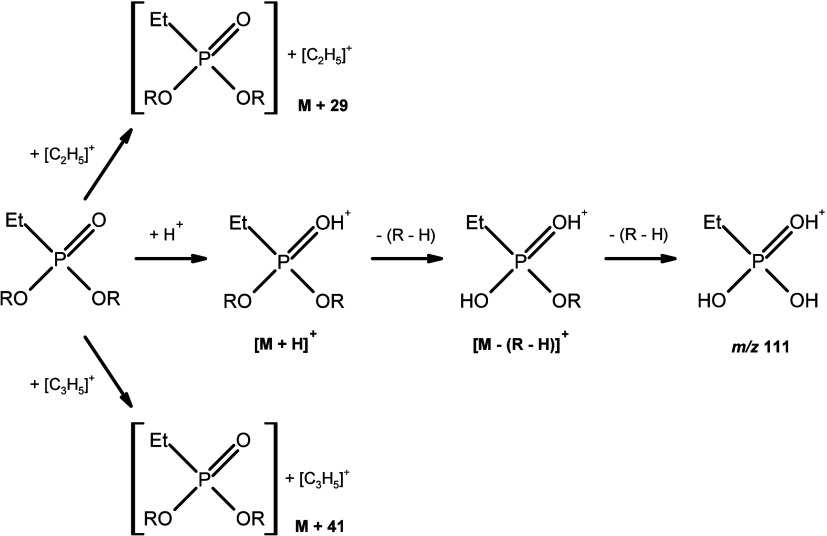
Probable mechanism for the formation of the main *O*,*O*-dialkyl ethylphosphonate ions in methane PCI. The figure was made based on literature information regarding the fragmentation of analytes in chemical ionization.^10,11^

Considering the results obtained during the analyzes of 32 representatives of symmetrical dialkyl ethylphosphonates and *O*-alkyl *O*-trimethylsilyl ethylphosphonates, the following regularities in the mass spectra can be presented, resulting from the similarities in the structure of compounds (Figures 3 and 6). The most important spectral and retention data are presented in Tables 1 and 2 for all 32 compounds.

**Figure 4:**
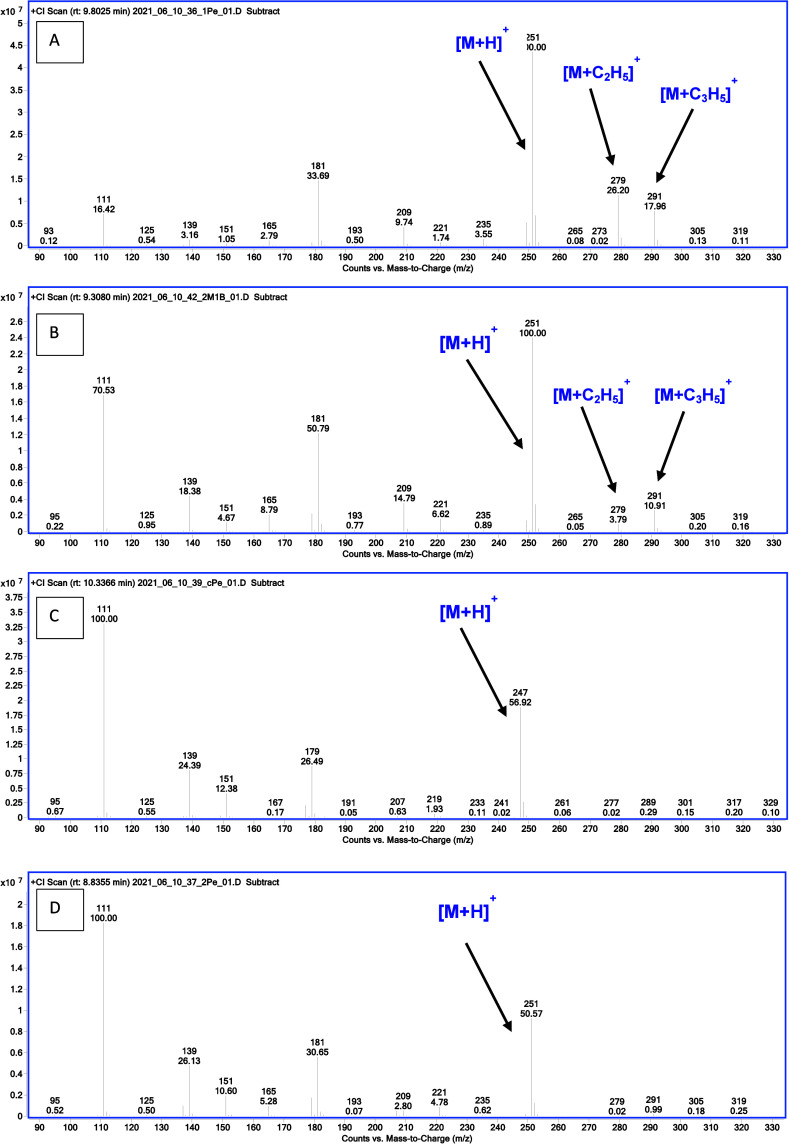
Comparison of PCI mass spectra of dialkyl derivatives of ethylphosphonic acid: (A) 1-pentyl, (B) 2-methyl-1-butyl, (C) cyclopentyl, and (D) 2-pentyl.

**Figure 5:**
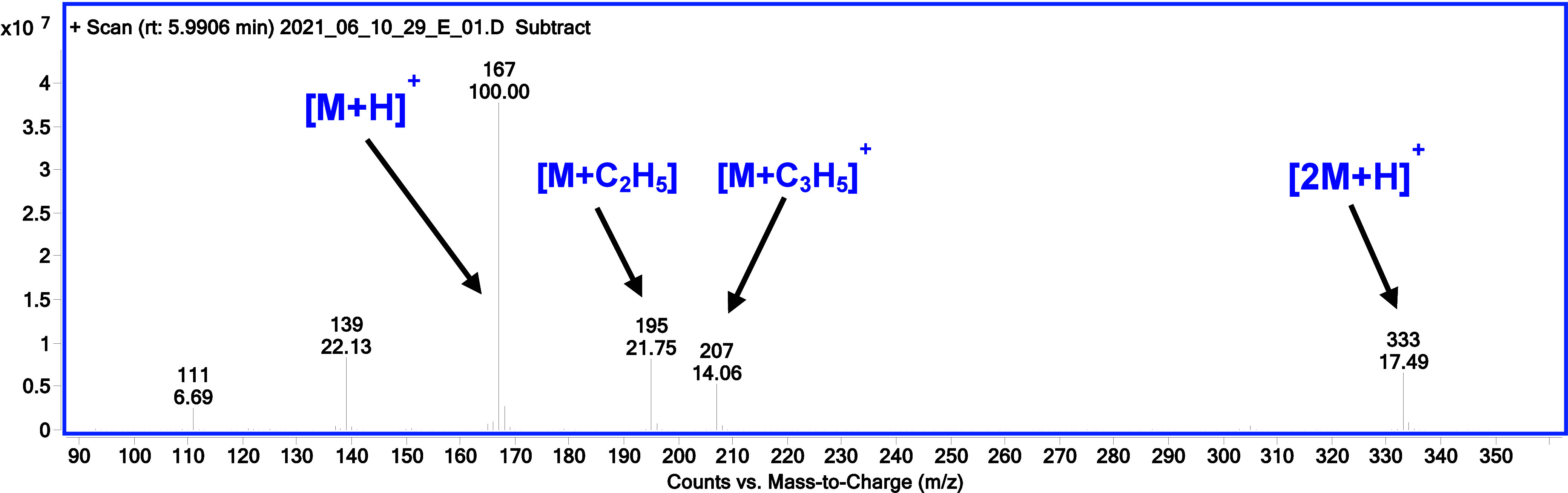
PCI mass spectra of diethyl ethylphosphonate confirming the formation of dimers (*m*/*z* = 333).

For diesters, the characteristic ion is *m*/*z* 111 and corresponds to the [C_2_H_8_PO_3_]^+^ fragment (Figure 3). Additionally, in the mass spectrum of esters we observe protonated molecule [M + H]^+^ and [M + C_2_H_5_]^+^, [M + C_3_H_5_]^+^ adducts (Figure 4A, B, and D). The exceptions are derivatives obtained by substitution of cycloalkyls. In this case, we do not observe [M + C_2_H_5_]^+^ and [M + C_3_H_5_]^+^ adducts (Figure 4C). For all diesters in mass spectra, we observe dimer ion [2M + H]^+^ (Figure 5). Monoesters are characterized by the presence of a pair of ions *m*/*z* 167 from [C_4_H_12_PO_3_Si]^+^ and 183 from [C_5_H_16_PO_3_Si]^+^ (Figure 7). In mass spectra of monoesters, we can observe protonated molecule [M + H]^+^ with high intensity, similarly to diesters, and we also observe [M + C_2_H_5_]^+^, [M + C_3_H_5_]^+^ adducts. Additionally, for monoesters, no dimers were found. For monoester in mass spectrum [M+73]^+^ ion occurs, as a result of the addition of the trimethylsilyl group (Si(CH_3_)_3_). The simultaneous observation of these ions in the CI spectrum may indicate the presence of ethylphosphonic acid derivatives in the tested samples. For unambiguous identification, it is advisible to supplement this type of data with data obtained from other techniques, especially using GC-EI-MS.

#### 3.2.1 *O*,*O*-Dialkyl Ethylphosphonates

(1) *N*-Alkyl derivatives: the protonated molecule [M + H]^+^ is the primary peak. [M + C_2_H_5_]^+^ and [M + C_3_H_5_]^+^ adducts are clearly visible and confirm the molecular weight of these compounds (Figure 4A).
(2) Other primary alkyl derivatives: the protonated [M + H]^+^ molecule is the base peak, but the [M + C_2_H_5_]^+^ and [M + C_3_H_5_]^+^ adducts are of lower intensity than in the *n*-alkyl derivatives. The *m*/*z* 111 ion, derived from the cleavage of both alkyl groups, has an intensity of 17 to 93% of the base ion. The ion formed as a result of the detachment of one alkyl group has an intensity greater than 40% of the base peak. Its *m*/*z* varies depending on the type of alkyl substituent introduced. In the case of branching at carbon γ, the course of fragmentation is similar to the fragmentation of *n*-alkyl derivatives (Figure 4B).
(3) Unsubstituted cycloalkyl derivatives: The adducts are barely visible. In the case of these derivatives, the *m*/*z* 111 ion dominates. The ion formed as a result of cleavage of the cycloalkyl group has an intensity of 5–55% of the base peak. The *m*/*z* value of the resulting fragment depends on the type of cycloalkyl substituent introduced (Figure 4C). The exception here is the dicyclobutyl derivative were the protonated molecule is the base peak and has an intensity of 0–o 61% of the base ion in the other derivatives.
(4) Secondary alkyl derivatives: the protonated molecule has an intensity of 5–75%, except for the di-2-propyl derivative where it is the primary ion. The spectra are dominated by the *m*/*z* 111 ion as the base ion (Figure 4D).
(5) Dimer ions appear [2M + H]^+^ (Figure 5).

#### 3.2.2 *O*-Alkyl *O*-Trimethylsilyl Ethylphosphonates

**Figure 6:**
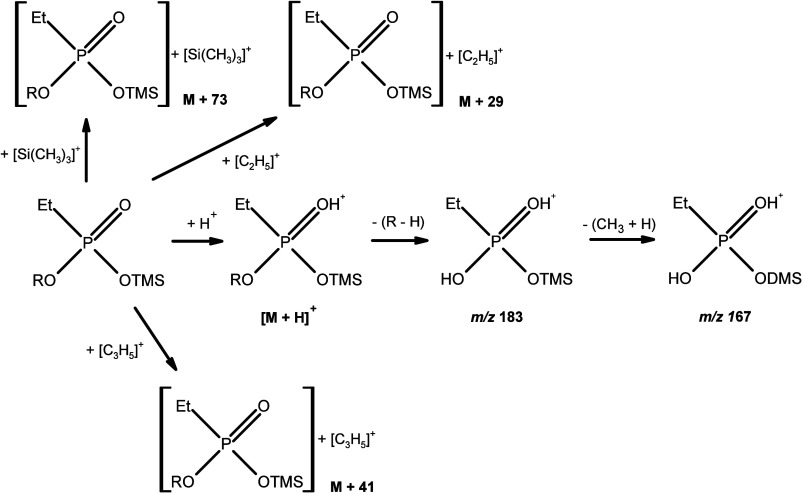
Probable mechanism for the formation of the main ions *O*-alkyl *O*-trimethylsilyl ethylphosphonates in methane PCI. The expression OTMS means: O–Si(CH_3_)_3_, and the expression ODMS means O–Si(CH_3_)_2_.

**Figure 7:**
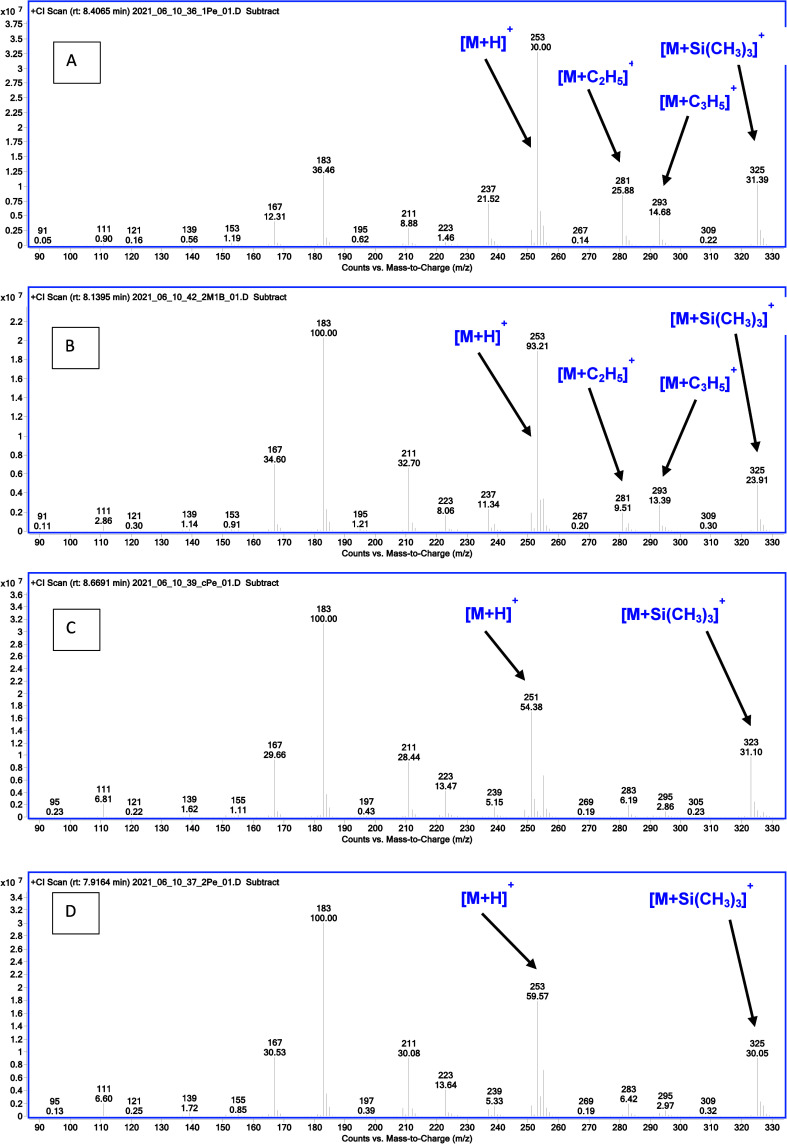
Comparison of PCI mass spectra of monoalkyl derivatives of ethylphosphonic acid: (A) 1-pentyl, (B) 2-methyl-1-butyl, (C) cyclopentyl, and (D) 2-pentyl.

Similar considerations apply to the second group of compounds obtained (Figure 6):

(1) *N*-Alkyl derivatives: the protonated [M + H]^+^ molecule is the primary peak. [M + C_2_H_5_]^+^ and [M + C_3_H_5_]^+^ adducts are clearly visible and confirm the molecular weight (Figure 7A).
(2) Other primary alkyl derivatives: the protonated molecule is the base peak for 2,2-dimethyl-1-propyl and 3-methyl-1-butyl derivatives. For the other derivatives, it has an intensity above 80% of the base peak. The adducts are visible (Figure 7B). The dominant *m*/*z* 183 ion is formed as a result of the cleavage of the alkyl group (Figure 6).
(3) Unsubstituted cycloalkyl derivatives: the protonated molecule is the base peak for the cyclobutyl derivative and has an intensity of 6 to 60% of the base peak for the other derivatives. The adducts are barely visible. The *m*/*z* 183 ion is dominant (Figure 7C).
(4) Secondary alkyl derivatives: the protonated molecule has an intensity of 12 to 75% of the base peak. In the pinacolyl derivative, the base ion is *m*/*z* 339 [M+73]^+^. The *m*/*z* 183 ion predominates as the base ion (Figure 7D).
(5) With an intensity of 0–100% there is an ion [M + 73]^+^; it is probably an adduct [M + Si(CH_3_)_3_]^+^ (which is shown in Figure 6).

### 3.3 Comparison of Retention Parameters

**Figure 8:**
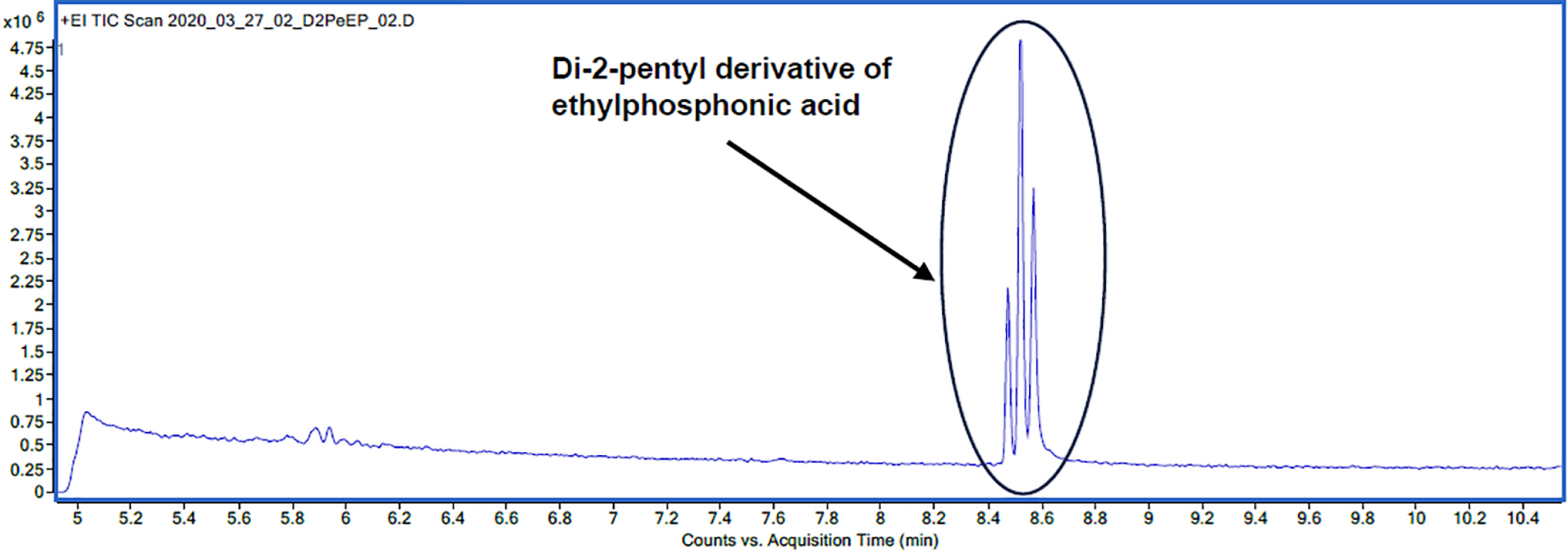
Chromatogram showing three peaks for the di-2-pentyl derivative of ethylphosphonic acid.

**Figure 9:**
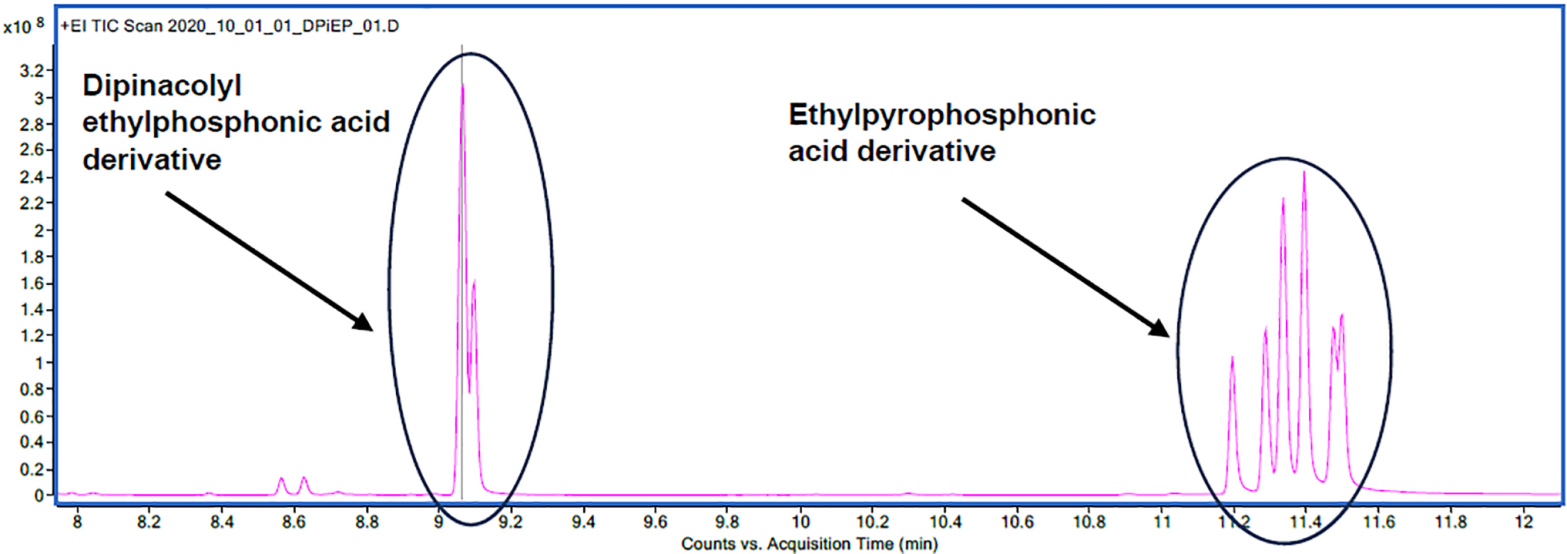
Chromatogram showing two peaks for the dipinacolyl ethylphosphonic acid derivative and a group of peaks for the ethylpyrophosphonic acid derivative.

As part of checking the results of the microsynthesis, analyses were carried out in the EI-MS mode toward the determination of retention indices, using the van den Dool and Kratz system, i.e., temperature programmed retention indices. The RI calculation was carried out automatically using the AMDIS program, using a mixture of C_7_–C_30_*n*-alkanes with a concentration of 10 ppm. Even though the stationary phase of the HP-5MS chromatographic column is not dedicated to the separation of optically active analytes, in the case of this class of alkyl diesters, three peaks were observed in some cases in the chromatograms (Figure 8). This may be additional information to support the identification process. In the case of ethylpyrophosphonates, which are byproducts of microsynthesis, instead of individual peaks, groups of peaks are observed (Figure 9).

An attempt to systematize the retention parameters is as follows. The relationship between *n*-alkyl and unsubstituted cycloalkyl derivatives is obvious.

To verify the correctness of the proposed series of homologues, the extrapolated retention index values were compared with the data from the OCADv24 library (Table 3).

**Table 3:** Comparison of Library Retention Indices to Those Calculated for Selected Derivatives

Lp.	Derivative	library RI	calculated RI	Difference: RI lib – RI cal
1	bis(2-heptyl) (Figure 10)	1828	1850	–22
2	mono 2-heptyl (Figure 10)	1529	1520	9
3	bis(2-octyl) (Figure 10)	2008	2024	–16
4	mono 2-octyl (Figure 10)	1618	1603	15
5	bis(4-methyl-1-pentyl) (Figure 14)	1768	1778	–10
6	mono 4-methyl-1-pentyl (Figure 14)	1483	1494	–11

Based on the example relationships shown in Table 3, it can be concluded that the suggested homologue series are correct and can support the identification process of this class of compounds. Additionally, the determined equations allow for determining the retention index for selected derivatives.

## 4 Conclusions

In order to carry out comparative studies, microsyntheses of 32 dialkyl derivatives of ethylphosphonic acid and the same amount of monoalkyl derivatives were performed. The synthesis yields toward diesters differed depending on the alcohol used. Unbranched n-alcohols were the most reactive, but their reactivity decreased with an increase in alkyl chain length. Primary branched alcohols and secondary (including cyclo) alcohols were less reactive. Microsyntheses were carried out to obtain symmetrical diesters; however, based on the analyzes carried out, it can be concluded that under these conditions (in some cases) also appropriate ethylpyrophosphonates were formed.

During the experiments conducted using chemical ionization, protonated analyte molecules with high relative intensities were obtained. In many cases, these are the base peaks in the spectrum. This is a definite advantage of this technique compared to electron beam ionization.

The obtained results allowed the group of synthesized compounds to depend on the structure of the introduced alkyl substituent. They were divided into derivatives of primary and secondary alcohols. Under the applied test conditions, *n*-alkyl diesters and other primary derivatives form protonated molecules, which are the base peaks in the CI mass spectrum. Secondary dialkyl derivatives (except cyclobutyl and 2-propyl) form protonated molecules of lower intensity. In the case of monoesters, the *n*-alkyl derivatives behave as in the case of diesters. However, only for the other two primary derivatives (2,2-dimethyl-1-propyl and 3-methyl-1-butyl), the protonated molecule is a primary peak. Secondary derivatives of monoesters behave similarly to diesters, except for the 2-propyl derivative.

For some compounds, it was not possible to obtain protonated molecules or their relative intensities were below 10%. The solution may be to change the operating parameters of the spectrometer, including by lowering the temperature of the ion source and the energy of the electron beam.

**Figure 10:**
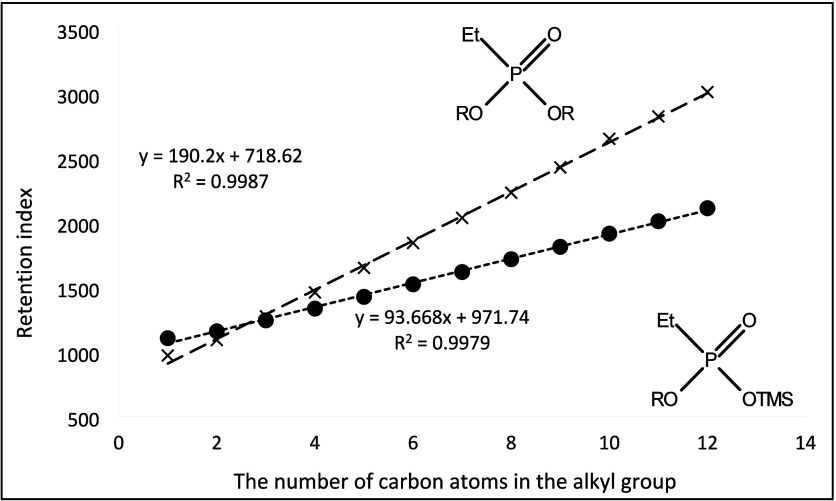
Dependence of the retention index of mono- and di-*n*-alkyl derivatives of ethylphosphonic acid on the number of carbon atoms in the *n*-alkyl substituent.

**Figure 11:**
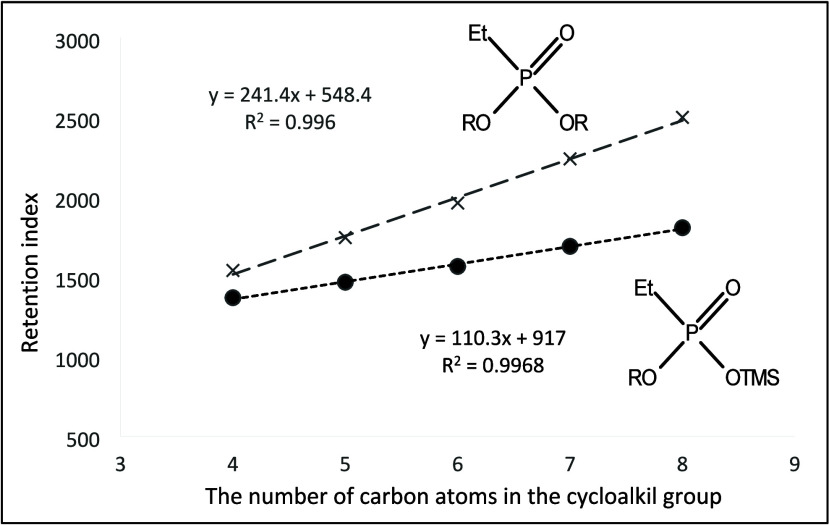
Dependence of the retention index of mono- and dicycloalkyl derivatives of ethylphosphonic acid on the number of carbon atoms in the cycloalkyl ring.

**Figure 12:**
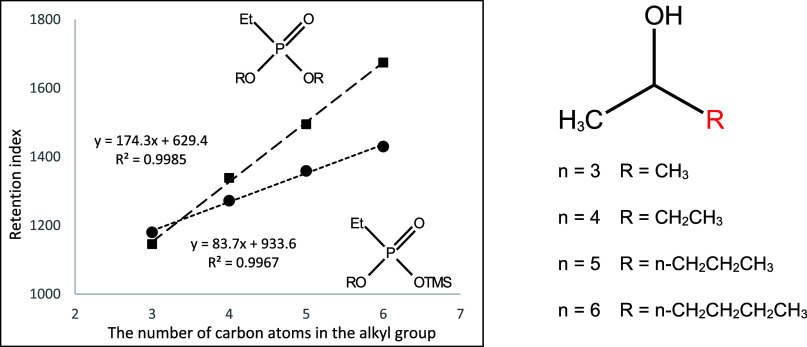
Dependence of the retention index of mono- and dialkyl derivatives of ethylphosphonic acid on the number of carbon atoms in the 2-alkenyl substituent.

**Figure 13:**
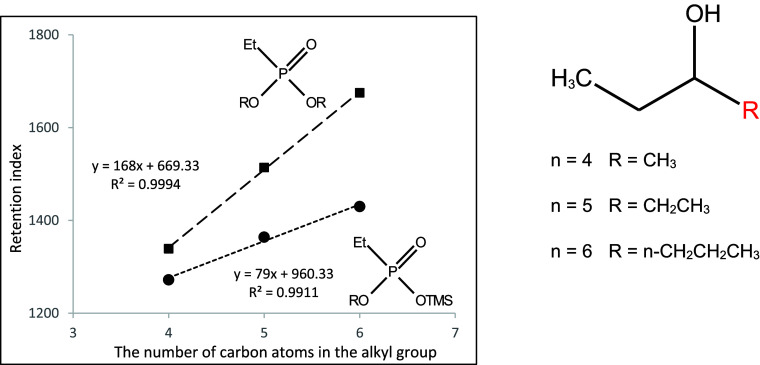
Dependence of the retention index of mono- and dialkyl derivatives of ethylphosphonic acid on the number of carbon atoms in the 1-alkylpropyl substituent.

**Figure 14:**
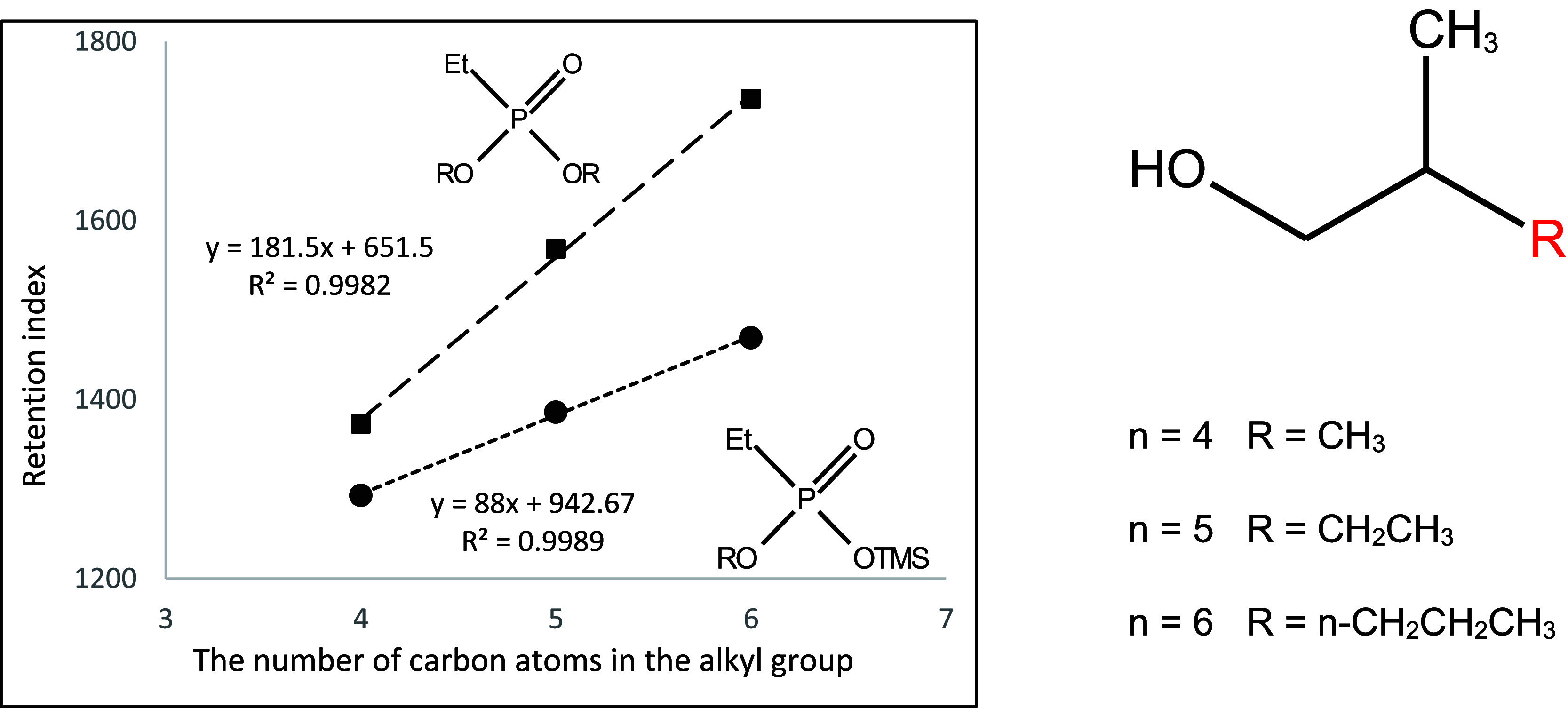
Dependence of the retention index of mono- and dialkyl derivatives of ethylphosphonic acid on the number of carbon atoms in the 2-alkylpropyl substituent.

**Figure 15:**
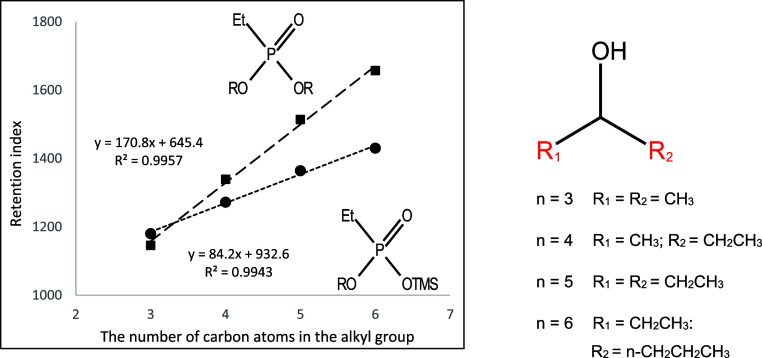
Dependence of the retention index of mono- and dialkyl derivatives of ethylphosphonic acid on the number of carbon atoms in the dialkylcarbinol substituent.

**Figure 16:**
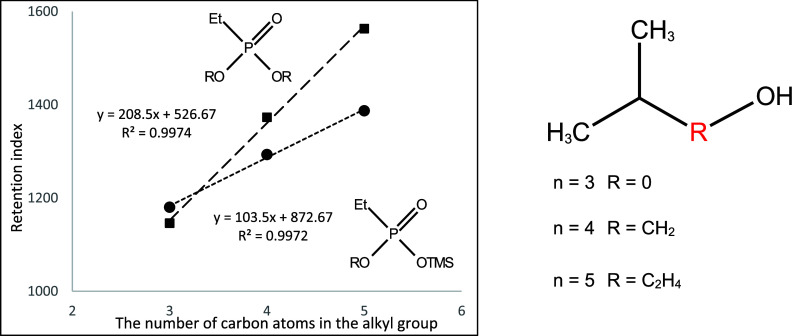
Dependence of the retention index of mono- and dialkyl derivatives of ethylphosphonic acid on the number of carbon atoms in the (ω – 1)-methylalkyl substituent.

**Figure 17:**
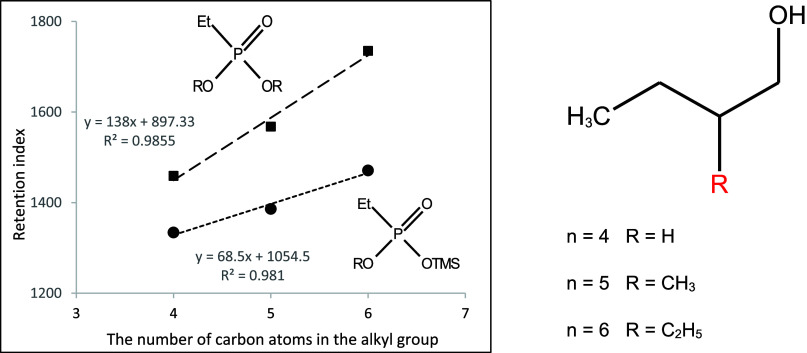
Dependence of the retention index of mono- and dialkyl derivatives of ethylphosphonic acid on the number of carbon atoms in the 2-alkylbutyl substituent.

Retention data of the tested analytes were collected during the experiments by using electron beam ionization. We observed regularities in the increase of retention indices for several series of homologues. They are shown in Figures 10–17. The presented relations can be used to estimate the retention parameters of subsequent higher representatives of the proposed series. They will also be used for comparison with data obtained during planned analogous studies with derivatives of other alkylphosphonic acids.

During the research, five compounds were synthesized, listed in the Introduction, whose spectra do not appear in EI mass spectral libraries, and their retention indices were not known. The identification of these substances was supported by the CI mass spectra and retention data using previously developed relationships.

The CI spectra of the 1-undecyl and 1-dodecyl derivatives are analogous to the spectral patterns of the lower *n*-alkyl homologues. In the case of both diesters and monoesters, protonated molecules are the base peak and there are [M + C_2_H_5_]^+^ and [M + C_3_H_5_]^+^ adducts. In diesters there are characteristic peaks *m*/*z* 111 and in monoesters there are characteristic peaks *m*/*z* 167 and 183. The retention indices of these compounds lie on characteristic trend lines, respectively, for both types of *n*-alkyl derivatives.

The spectral data of the next synthesized compound, *O*-cyclobutyl *O*-TMS ethylphosphonate, retain the rule of its higher homologues, so there is a high-intensity protonated molecule peak and characteristic peaks *m*/*z* 167 and 183. The retention index correlates with the trend of the unsubstituted cycloalkyl derivatives.

Therefore, it is reasonable to conclude that the research method used is useful and effective.
